# Synergistic Effect of Mitochondrial and Lysosomal Dysfunction in Parkinson’s Disease

**DOI:** 10.3390/cells8050452

**Published:** 2019-05-14

**Authors:** Flora Guerra, Giulia Girolimetti, Raffaella Beli, Marco Mitruccio, Consiglia Pacelli, Anna Ferretta, Giuseppe Gasparre, Tiziana Cocco, Cecilia Bucci

**Affiliations:** 1 Department of Biological and Environmental Sciences and Technologies (DiSTeBA), University of Salento, Via Provinciale Lecce-Monteroni 165, 73100 Lecce, Italy; raffaella.beli@unisalento.it (R.B.); marco.mitruccio@unisalento.it (M.M.); 2 Department of Medical and Surgical Sciences (DIMEC), Medical Genetics Unit, University of Bologna, via Massarenti 9, 40138 Bologna, Italy; giulsgiuls85@gmail.com (G.G.); Giuseppe.gasparre3@unibo.it (G.G.); 3 Department of Clinical and Experimental Medicine, University of Foggia, 71122 Foggia, Italy; consiglia.pacelli@unifg.it; 4 Department of Basic Medical Sciences, Neurosciences and Sensory Organs, University of Bari “A. Moro”, 70122 Bari, Italy; annaferretta@tiscali.it (A.F.); tizianamaria.cocco@uniba.it (T.C.)

**Keywords:** Parkinson’s disease, lysosome, autophagy, endocytosis, mitochondria

## Abstract

Crosstalk between lysosomes and mitochondria plays a central role in Parkinson’s Disease (PD). Lysosomal function may be influenced by mitochondrial quality control, dynamics and/or respiration, but whether dysfunction of endocytic or autophagic pathway is associated with mitochondrial impairment determining accumulation of defective mitochondria, is not yet understood. Here, we performed live imaging, western blotting analysis, sequencing of mitochondrial DNA (mtDNA) and senescence-associated beta-galactosidase activity assay on primary fibroblasts from a young patient affected by PD, her mother and a healthy control to analyze the occurrence of mtDNA mutations, lysosomal abundance, acidification and function, mitochondrial biogenesis activation and senescence. We showed synergistic alterations in lysosomal functions and mitochondrial biogenesis, likely associated with a mitochondrial genetic defect, with a consequent block of mitochondrial turnover and occurrence of premature cellular senescence in *PARK2*-PD fibroblasts, suggesting that these alterations represent potential mechanisms contributing to the loss of dopaminergic neurons.

## 1. Introduction

Parkinson’s disease (PD) is one of the most common neurodegenerative disorders, second only to Alzheimer’s disease. PD is characterized by motor symptoms as bradykinesia, resting tremor, postural instability, and muscle rigidity together with non-motor signs such as anosmia, sleep disorders, depression and, with disease progression, dementia. The principal features of PD are the selective loss of dopaminergic neurons in the substantia nigra pars compacta and the presence of protein aggregates (e.g., Lewy bodies, LB) in remaining neurons post mortem [1].

The pathological mechanisms of PD are largely unknown, although it has been established that multifactorial and genetic causes are involved. Associated with exposure to environmental toxins [2], most PD cases are sporadic, while only about 10% are familial, frequently of early onset [3] and correlated with monogenic mutations in 15 causative genes. Among these, mutations in the leucine-rich repeat kinase 2 gene (*LRRK2*) [4] or in the α-synuclein gene [5] are causes of PD autosomal dominant forms, whereas genes involved in autosomal recessive PD include parkin [6], PTEN-induced putative kinase 1 (*PINK1*) [7], *DJ-1* [8] and *ATP13A2* [9]. Moreover, mutations in many other genes, identified through genome-wide association studies, correlate with increased risk to develop the disease. Interestingly, most of the proteins encoded by these genes are implicated in mitochondrial quality control pathways, varying from mitochondrial proteins to proteins regulating endo-lysosomal function [10]. 

Several studies have demonstrated impairment of mitochondrial respiratory complex I (CI) function in in vivo and in vitro models of PD, as well as in human parkinsonism due to intoxicants [11,12]. Environmental exposure to neurotoxin 1-methyl-4-phenyl- 1,2,3,6-tetrahydropyridine (MPTP), an inhibitor of mitochondrial CI, determines depletion of ATP production, Reactive Oxygen Species (ROS) production, degeneration of dopaminergic neurons and parkinsonism [13]. Also, mitochondrial dysfunction and neurotoxicity are caused by transport of herbicide paraquat, which is reduced by NADPH oxidase in microglia, into dopaminergic neurons [14]. Moreover, rotenone, a well-established CI inhibitor, is a pesticide that induces parkinsonian phenotype in animal models [15], and environmental exposure to this compound may increase the risk of PD also in humans [2].

Interestingly, mitochondrial dysfunction was also induced by PD-linked mutations [16,17]. Indeed, dysfunction of CI, dissipation of mitochondrial membrane potential, disruption of Ca^2+^ homeostasis, and enhanced release of cytochrome *c* were observed in cellular and animal models with soluble prefibrillar α-synuclein oligomers [16]. 4-hydroxynonenal, a lipid peroxidation product, promotes, in an in vitro model of PD, the accumulation of α-synuclein aggregates and the extrusion of extracellular vesicles (EVs) containing toxic α-synuclein [18]. Internalization of these EVs into neighboring neurons causes their degeneration finally resulting in the development of PD [18]. Mitochondrial fragmentation and neuronal death were observed also in PD patients with mutations in the Vacuolar Protein Sorting 35 (*VPS35*) gene, encoding a key component of the retromer complex which regulates the process of transmembrane protein sorting between endosomes and the Golgi [19]. Consistently, VPS35 deficiency too leads to mitochondrial dysfunction and finally results in the loss of dopaminergic neurons [20]. Glucocerebrosidase (*GBA1*) gene mutations are the most common genetic risk factor for PD [21]. Glucocerebrosidase 1 (GCase 1) catabolizes the glycolipid glucocerebroside to ceramide and glucose in lysosomes [22]. Interestingly, inhibition of GCase 1 enzymatic activity, or *GBA1* silencing causes impairment of mitochondrial function in SH-SY5Y, with deficit in the mitochondrial respiratory chain activity, mitochondrial depolarization and fragmentation, and elevated levels of ROS [23].

Notably, the familial forms of PD associated with mutations in genes important in the regulation of the autophagic–lysosomal pathway often show mitochondrial deficit [20,24,25,26]. In fact, α-synuclein aggregation and *LRRK2* mutations determine, through different mechanisms, dysregulation of autophagic and endo-lysosomal pathways, but also mitochondrial dysfunction [27,28,29,30]. On the other hand, a rapid increase in the transcriptional level of a number of lysosomal genes was induced by acute exposure of mouse embryonic fibroblasts to rotenone, while a marked decrease in the expression of the same genes was caused by chronic treatment [31].

What emerges from the knowledge obtained so far on the molecular mechanisms of non-idiopathic PD pathogenesis is that the crosstalk between lysosomes and mitochondria plays a central role. Indeed, both parkin and PINK1 are involved in the mitophagy process, needed for clearance of dysfunctional mitochondria [32]. Mitophagy is activated by mitochondrial damage following by PINK1 stabilization on the outer mitochondrial membrane, direct PINK1 phosphorylation and mitochondrial recruitment of parkin. Activated parkin, which is a multifunctional E3 ubiquitin ligase, polyubiquitinates mitochondrial proteins, leading to their association with the ubiquitin-binding domains of autophagy receptors, causing the formation of the autophagosome, its subsequent fusion with lysosomes and, finally, mitochondrial autophagic degradation [33].

Lysosomal dysfunction and enlargement of the lysosomal compartment is induced by PINK1 depletion [34]. In addition, inhibition of the mitochondrial ATP-synthase using oligomycin [34] and knockout of TFAM, the major transcription factor for mitochondrial biogenesis determine lysosomal compartment defects [35]. Furthermore, the PD-related protein DJ-1, localized to mitochondria [36,37], is involved in both mitochondrial function and autophagy. DJ-1 silencing in M17 neuroblastoma cell line causes a reduction of mitochondrial membrane potential, mitochondrial fragmentation and accumulation of autophagy markers [38]. 

Altogether, these data suggest that in PD lysosomal function may be influenced by mitochondrial quality control, dynamics and/or respiration. However, whether dysfunction of the autophagy–lysosomal pathway is associated with mitochondrial impairment determining accumulation of defective mitochondria through failed mitophagy/autophagy, or other pathways, has not been clarified. 

Mutations in parkin gene (*PARK2*) cause inherited juvenile parkinsonism and since these mutations were discovered, about half of all Parkinson’s disease cases have been correlated to mutations in this gene. 

In this work, primary fibroblasts from a patient affected by a juvenile form of PD with a compound heterozygous deletional mutation of *PARK2* gene, previously used to characterize mitochondrial dysfunction [39], were studied. We showed synergistic alterations in lysosomal function and in mitochondrial biogenesis. We concluded that this scenario, likely associated with mitochondrial genetic defects and characterized by block of mitochondrial turnover and occurrence of premature cellular senescence, could be one of the mechanisms contributing to the loss of dopaminergic neurons. 

## 2. Materials and Methods

### 2.1. Skin Fibroblasts and Culture Conditions

Primary fibroblasts from a young patient affected of PD juvenile form (*PARK2*-PD, 36 years old), the mother (parental healthy control, CTR2, 59 years old) [39] and a healthy control (CTR1, 46 years old) [40], were obtained by explants from skin punch biopsy, after informed consent, as previously described. CTR2 and *PARK2*-PD cells were previously described and indicated as CTRL and P1, respectively [39]. *PARK2*-PD cells carry a compound heterozygous deletional mutation of *PARK2* (del exon2-3/del exon3), while the CTR2, unaffected parental control, displays the heterozygous del exon2-3 [39]. Cells were grown in high-glucose Dulbecco’s modified Eagle’s medium (DMEM, Corning, NY, USA) supplemented with 20% (*v*/*v*) fetal bovine serum (FBS), 1% (*v*/*v*) L-glutamine, 1% (*v*/*v*) penicillin/streptomycin (Sigma-Aldrich, St. Louis, MO, USA) at 37 °C in a humidified atmosphere of 5% CO_2_. All experiments were performed on cells with similar passage numbers. For starvation in nutrient-depleted medium, cells were incubated in Earle’s Balanced Salt Solution (EBSS) (Sigma-Aldrich, Saint Louis, MO, USA) for 3 h at 37 °C in a humidified atmosphere of 5% CO_2_. 

### 2.2. NSC34 Cells and Culture Conditions

The mouse motoneuron-like hybrid cell line NSC34 [41] was cultured in Dulbecco’s modified Eagle’s medium (DMEM, Corning, NY, USA) supplemented with 10% (*v*/*v*) fetal bovine serum (FBS), 1% (*v*/*v*) L-glutamine, 1% (*v*/*v*) penicillin/streptomycin (Sigma-Aldrich, St. Louis, MO, USA) at 37 °C in a humidified atmosphere of 5% CO_2_. Rotenone (Santa Cruz Biotechnologies, Dallas, TX, USA), an inhibitor of mitochondrial complex I, used to induce Parkinson-like syndrome as an experimental model in rats, was added to the culture medium at 100 nM for 24 h.

### 2.3. Immunofluorescence and Live Microscopy

For immunofluorescence analysis cells grown on 11 mm round glass coverslips were permeabilized with piperazine-N,N′-bis(2 ethanesulfonic acid) (PIPES)-Saponin 0.1% buffer for 2 min, fixed in 3% paraformaldehyde for 20 min, washed in Phosphate Buffer Saline (PBS)-Saponin 0.1%, treated with NH_4_Cl (50 mM) for 10 min and incubated with the primary antibody in PBS- Saponin 0.1% for 20 min. After 3 washes with PBS-Saponin 0.1%, samples were incubated for 20 min with the secondary antibody in PBS-Saponin 0.1%, washed three times with PBS-Saponin 0.1% and finally rinsed in PBS 1×. Coverslips were then mounted on a drop of Mowiol (Calbiochem-Novabiochem Corporation, La Jolla, CA, USA). For LAMP1 staining we used mouse anti-LAMP1 (H4A3), deposited to the Developmental Studies Hybridoma Bank (University of Iowa, Iowa City, IA, USA) by J.T. August and J.E.K. Hildreth at 1:250 dilution, and goat anti-mouse AlexaFluor488 (ThermoFisher, Carlsbad, CA, USA) at 1:500 dilution. 

For live microscopy, cells were seeded into microscopy chambers (8 well μ-slide, Ibidi GmBh, Martinsried, Germany) and, after 24 h, incubated with 1 μM LysoTracker Red DND-99 for 30 min at 37 °C, 1 μM LysoSensor Blue DND-167 or 1 μM LysoSensor Yellow/Blue DND 160 for 5 min at 37 °C. These dyes were from ThermoFisher Scientific (Carlsbad, CA, USA) and are characterized by amine groups, which are partially protonated at neutral pH and fully protonated at acidic pH. In particular, LysoTracker DND-99 fluorescence is somewhat pH-independent, while LysoSensor DND-160 and DND-167 are able to detect compartments within the pH range of 3.5–6.0 and 4.5–6.0, respectively [42]. After 3 washes in PBS, L-15 medium (Leibowitz medium without phenol red, Gibco, ThermoFisher) was added and the cells were imaged by confocal microscopy. We used also MitoTracker Green FM (500 nM) or MitoTracker Red CM-H2XROS (500 nM) (ThermoFisher Scientific) as described previously [43]. Briefly, cells were incubated with the dyes for 45 min at 37 °C in DMEM medium without serum. After 3 washes in PBS, L-15 medium was added and cells were imaged by confocal microscopy. 

Fluorescence images were captured using a confocal laser scanning microscope (CLSM) (Zeiss, LSM 700, Germany) equipped with a laser diode emitting at 405 nm, an argon-ion laser for excitation at 488 nm, and a helium-neon laser for excitation at 555 nm. 

Emission intervals for individual dyes were: AlexaFluor488: 495–550 nm (λ_ex_ = 488 nm); LysoTracker DND-99: 560–615 nm (λ_ex_ = 555 nm); LysoSensor DND-167: 415–500 nm (λ_ex_ = 405 nm); LysoSensor DND-160: 500–590 nm (λ_ex_ = 405 nm); MitoTracker Green FM: 495–550 nm (λ_ex_ = 488 nm) and MitoTracker Red CM-H2XROS: 560–615 nm (λ_ex_ = 555 nm). Images were taken with a Plan-Apochromat 63.0 × 1.40 oil- immersion objective DIC M27 and the pinhole aperture was set to 1 Airy unit. The images were acquired using ZEN Black Edition 2011 software (Zeiss, Jena, Germany).

Intensity of fluorescence was determined by ImageJ software (Version 1.5Oi, Bethesda, MD, USA) and it was calculated as intensity/cell normalizing on the area of each single cell. Measures were obtained by analyzing at least 50 cells/sample in at least three independent experiments. 

### 2.4. DQBSA (Self-Quenched BODIPY Dye Conjugates of Bovine Serum Albumin) Assay

Cells were seeded on 11 mm round glass coverslips and incubated in the presence of Red DQ-BSA (10 μg/mL) or Green DQ-BSA (50 μg/mL) (ThermoFisher Scientific, Carlsbad, CA, USA) for 24 h (for NSC34 Cells) or 48 h (for fibroblasts) at 37 °C with full medium in a humidified atmosphere of 5% CO_2_. Subsequently, cells were fixed in methanol, incubated with DAPI dye (1 μg/mL) and analyzed with LSM 700 confocal microscope (Zeiss). DAPI was excited with 405 nm laser diode and emission was collected from 405 nm to 490 nm. Emission intervals: Red DQBSA = 560–615 nm (λ_ex_ = 555 nm) and Green DQBSA = 495–550 ((λ_ex_ = 488 nm). Images were taken with a Plan-Apochromat 63.0 × 1.40 oil- immersion objective DIC M27 and the pinhole aperture was set to 1 Airy unit. Images were acquired using ZEN Black Edition 2011 acquisition software (Zeiss, Germany).

Intensity of fluorescence was determined by ImageJ software (Version 1.5Oi, Bethesda, MD, USA). Intensity of fluorescence was calculated as intensity/cell normalizing on the area of each single cell. Measures were obtained by analyzing at least 50 cells/sample for at least three different experiments. 

### 2.5. Western Blotting

Cells were lysed in Laemmli Buffer (100 mM Tris–HCl, pH 6.8, containing 4% SDS, 20% glycerol and 0.2% blue bromophenol). The protein amount in cell extracts was quantified loading extracts on Coomassie Blue gel (12% SDS-PAGE) together with a control lysate of known concentration and using ImageJ software to quantify. On each well of SDS-PAGE gels about 20 to 50 μg of lysates were loaded. Western blotting was performed as described [44]. Antibodies against RAB7A, ATP6V1G1, TFAM, Cathepsin D, GAPDH were from Santa Cruz Biotechnology (Dallas, TX, USA). Antibody against LC3 was from Nanotools (GmbH and Co. KG, Teningen, Germany), anti-p62 from BD Biosciences (San Jose, CA, USA), anti-p21 and anti-LAMP1 from Abcam (Cambridge, MA, USA). We used gels at 12% of acrylamide to detect RAB7A and p62, at 15% of acrylamide to detect ATP6V1G1, TFAM, LC-3, p21 and at 10% of acrylamide to detect Cathepsin D and LAMP1. Anti-α-tubulin was from Sigma-Aldrich (St Louis, MO, USA). Secondary anti-mouse and anti-rabbit antibodies, conjugated with HRP, were from Biorad (Hercules, CA, USA). Bands were detected using Clarity Max TM Western ECL substrate (Biorad) and UltraCruz Autoradiography (X-ray) film (Santa Cruz Biotechnology). Briefly, polyvinylidene difluoride membrane (PVDF) was blocked in Milk 5% in PBS/Tween 0.1% for 1 h, incubated with primary antibody in 5% defatted milk in PBS-Tween 0.1% for 1 h, washed three times in PBS/Tween 0.1%, incubated with secondary antibody in defatted Milk 5% in PBS/Tween 0.1% for 30 min and washed three times with PBS-Tween 0.1%. Substrate kit components were mixed 1:1 and membrane was incubated for 5 min before of exposure in X-Ray film cassette. Images were then acquired with Epson Perfection V600 Scanner (Epson, Suwa, Japan) and bands were quantified by densitometry using ImageJ software (Version 1.5Oi, Bethesda, MD, USA) normalizing against α-tubulin or GAPDH. 

#### 2.5.1. Maturation of Cathepsin D

Cathepsin D is a lysosomal protease synthesized as preprocathepsin D precursor, converted into procathepsin D (52 kDa) in the endoplasmic reticulum, and further processed in the acidic milieu of late endosomes and lysosomes, into the 44-kDa form and finally into the 32-kDa mature form [45]. Maturation of Cathepsin D was evaluated through western blotting analysis with an anti-Cathepsin-D antibody (Santa Cruz Biotechonolgy) that recognizes, not only the 32 kDa mature form, but also the 52 kDa and 44-kDa immature forms. Protein levels were quantified by densitometry with ImageJ software and amount of maturation was expressed as ratio between immature forms (52/44 kDa) and mature form (32 kDa). 

#### 2.5.2. Autophagic Flux

To examine the autophagic flux, we determined if LC3 is degraded in a lysosomal-dependent manner by using Bafilomycin A, an inhibitor of lysosomal acidification but also, independently of its effect on lysosomal pH, of fusion between autophagosomes and lysosomes [46,47,48]. To this purpose, fibroblasts were incubated with full medium (untreated) or with medium containing Bafilomycin A (400 nM for 3 h). Western blot analysis was performed on cell lysates using antibodies against LC3 (Nanotools) and α-tubulin (Sigma-Aldrich). The amount of LC3 II was calculated normalizing on α-tubulin, through densitometric analysis using ImageJ software, and the autophagic flux was calculated as the ratio of LC3II of cells treated with Bafilomycin A and untreated.

### 2.6. Nucleic Acid Extraction and Whole mtDNA Amplification, Sequencing and Mutation Screening

Whole genomic DNA was extracted using Mammalian Genomic DNA Miniprep Kit (Sigma-Aldrich) according to the manufacturer’s protocols. Sanger sequencing of the entire mtDNA was performed following a quality-check protocol as previously described [49]. Mitochondrial DNA mutations were confirmed using a second PCR reaction. In silico prediction of the pathogenic potential of missense mutations was performed with PolyPhen2 (http://genetics.bwh.harvard.edu/pph2/) as previously described [50]. FASTA files were used as input for MToolBox [51] in order to annotate mitochondrial variants and related features, which read mapping, post-mapping processing, genome assembly, haplogroup prediction and variant annotation. Nucleotide site-specific variability was estimated on the multi-alignment of the updated healthy genomes reported in HmtDB [52] and in HmtVar [53].

### 2.7. Senescence-Associated Beta-Galactosidase (SA-βgal) Activity Assay

The SA-β-Gal activity assay was performed as previously described [54]. Briefly, cells were seeded in 6-well plates and, after 24 h, washed twice with PBS and then incubated for 7 min with a fixation solution (2% formaldehyde and 0.2% glutaraldehyde). Cells were then washed five times with PBS and incubated with a staining solution (40 mM citric acid/Na phosphate buffer pH 6, 1 mg/mL X-gal, 5 mM K4[Fe(CN)6]3H2O, 5 mM K3[Fe(CN)6], 150 mM NaCl and 1 mM MgCl_2_ in ddH2O) at 37 °C overnight or until β-Gal staining became visible. Cells were finally washed five times with PBS and once in methanol. Image acquisition and cell counting was performed using EVOS FL Auto Cell Imaging System (ThermoFisher) with UPlanFLN 4 × 0.13 PhP (Olympus, Shinjuku, Tokyo, Japan).

### 2.8. Statistical Analysis

All experiments were repeated at least three times and represented as mean ± standard error (SE). Statistical significance was determined for all experiment through Student’s t test for unpaired data (* *p* ≤ 0.05, ** *p* ≤ 0.01 and *** *p* ≤ 0.001).

## 3. Results

### 3.1. PARK2-PD Fibroblasts Display Abnormal Abundance, Acidification and Morphology of the Late Endocytic Compartment

Previous data obtained on *PARK2*-PD fibroblasts harboring a compound heterozygous deletion (del exon2-3/del exon3) in *PARK2* displayed mitochondrial defects and Peroxisome proliferator-activated receptor Gamma Coactivator 1-alpha (PGC-1α) dysfunction [39], severe ultrastructural abnormalities, mainly in mitochondria and cytoskeleton [39,55], and altered expression level of several proteins involved in cytoskeleton structure dynamics, Ca^2+^ homeostasis, oxidative stress response protein and RNA processing [56,57]. Considering the cross-talk between lysosomes and mitochondria in PD and that lysosomal function can be influenced by mitochondrial activity [58], we decided to monitor lysosomal morphology, size, distribution and activity.

**Figure 1:**
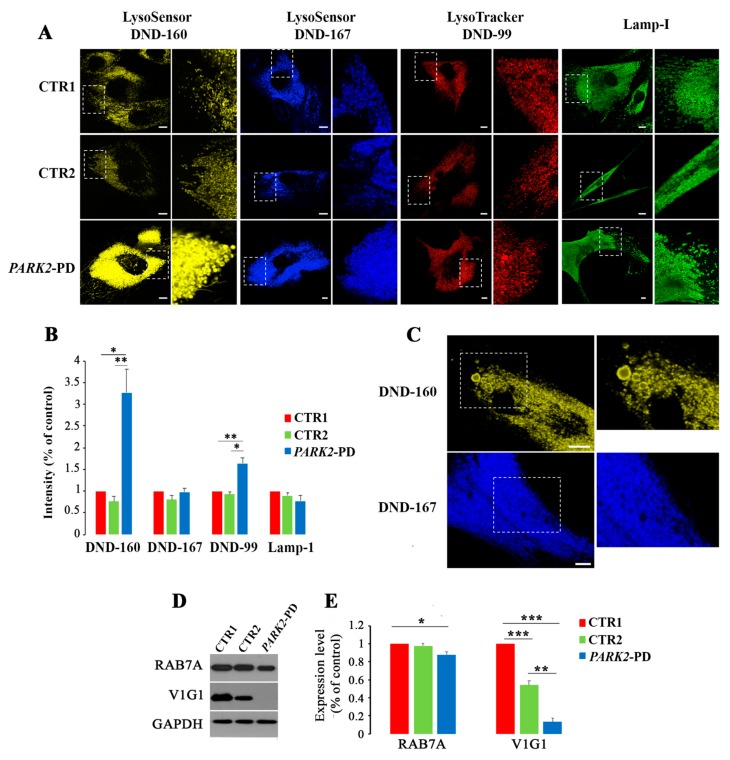
Alterations of late endocytic compartments in *PARK2*-PD cells. (**A**) Cells were labeled live with LysoSensor DND-160 (yellow), with LysoSensor DND-167 (blue) and with Lysotracker Red DND-99 or were fixed and immunostained with anti-LAMP1 antibody (green). White dashed boxes indicate zoomed areas on the right. Scale bar: 10 μm. (**B**) Immunofluorescence intensity was quantified by ImageJ software. Data represent the mean ± s.e.m. (three independent experiments, ≥ 20 cells). (**C**) Staining with LysoSensor DND-160 (yellow) and with LysoSensor DND-167 (blue) show enlarged lysosomes in *PARK2*-PD cells. White dashed boxes indicate zoomed areas on the right. Scale bar: 10 μm. (**D**,**E**) Relative protein abundance of RAB7A and ATP6V1G1 was assessed by western blotting and quantified by densitometry normalizing against GAPDH. Data represent the mean ± SE of at least three independent experiments (* *p* ≤ 0.05, ** *p* ≤ 0.01 *** *p* ≤ 0.001).

We started by monitoring intracellular distribution of lysosomes in healthy control cells (CTR1), in unaffected parental control cells (CTR2) and in patient’s fibroblasts (*PARK2*-PD) through live imaging microscopy using specific vital dyes to stain acidic organelles such as LysoTracker DND-99, LysoSensor DND-160 and LysoSensor DND-167 (Figure 1A). As shown in Figure 1A, *PARK2*-PD cells displayed a significant increase of acidic compartments stained with LysoSensor DND-160 or with LysoTracker DND-99 and intensity quantification revealed about 3 and 1.5-fold change, respectively, for dyes staining in *PARK2*-PD cells compared to control cell lines (Figure 1B). Instead, staining with LysoSensor DND-167 did not reveal a significant difference in the abundance of labeled organelles (Figure 1A,B) suggesting that only lysosomes with lower pH are affected, considering the pH range of detection of the dye. Similarly, immunofluorescence analysis using antibodies against the lysosomal-associated membrane protein 1 (LAMP-1), a marker of late endosomes and lysosomes, did not reveal any significant increase. Interestingly, we also observed altered lysosomal morphology with abnormally large peripheral organelles in *PARK2*-PD cells (Figure 1C). 

RAB7A is a small GTPase with a central role in the late endocytic pathway [59,60,61]. It is pivotal in controlling the early and late endosome maturation, lysosomal biogenesis and acidification, but also clustering and fusion of late endosomes and lysosomes in the perinuclear region [61,62]. Moreover, RAB7A regulates pH of endocytic organelles by controlling assembly and activity of the vacuolar ATPase (V-ATPase) on RAB7A-positive organelles through the interaction of the RAB7A effector RILP (Rab Interacting Lysosomal Protein) with the ATP6V1G1 subunit of the V-ATPase [63,64].

Considering the RAB7A role in regulating lysosomal biogenesis and functions, as well as lysosomal pH controlling V-ATPase assembly and activity, we performed western blotting analysis to evaluate the expression level of RAB7A and of the ATP6V1G1 subunit of V-ATPase (Figure 1D). We found a small but significant RAB7A reduction (10%) in *PARK2*-PD compared to CTR1 and about 80% and 40% reduction of ATP6V1G1 compared to CTR1 and CTR2, respectively (Figure 1D,E). 

Altogether these results indicate that *PARK2*-PD cells are characterized by altered acidification of endocytic compartments confirmed by downregulation of ATP6V1G1 expression.

### 3.2. Lysosomal Function Is Impaired in PARK2-PD Fibroblasts

With the aim of understanding if altered acidic compartments in *PARK2*-PD fibroblast were dysfunctional, we decided to perform a DQBSA assay to detect lysosomal proteolytic activity [65]. Indeed, this dye is strongly self-quenched and digestion of the BSA conjugates results in dequenching as the dye-labeled released protein fragments are brightly fluorescent. Moreover, DQBSA is insensitive to pH from pH 3–11 allowing the direct detection of proteolytic activity in situations where the pH is unknown and cannot be controlled or where the pH is known to be low (e.g., lysosomes and endosomes).

**Figure 2:**
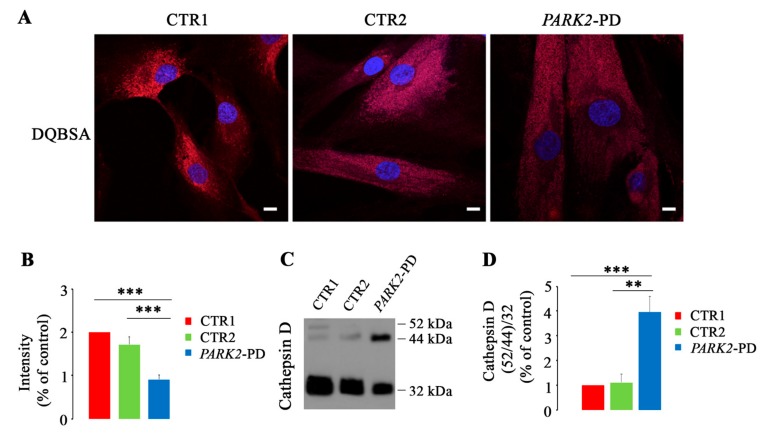
Alterations of lysosomal activity in *PARK2*-PD cells. (**A**,**B**) Cells were incubated in the presence of Red DQ-BSA and immunofluorescence intensity was quantified by ImageJ software. Nuclei were labeled with DAPI (blue). Scale bar: 10 μm. (**C**,**D**) Relative abundance of three Cathepsin D forms was assessed by western blotting and quantified by densitometry normalizing against 32 kDa mature form. (Data represent the mean ± SE of at least three independent experiments (* *p* ≤ 0.05, **p ≤ 0.01 *** *p* ≤ 0.001).

As shown in Figure 2A, control cells had similar proteolytic activity, as measured with the DQBSA assay although CTR2 (the unaffected mother of the proband) had lower values. In contrast, patient’s fibroblasts were characterized by a significant reduction (55% and 40%) of DQBSA intensity compared to CTR1 and CTR2 respectively, indicating that these cells display strongly decreased proteolytic activity of late endocytic organelles (Figure 2B). 

Cathepsin D is an aspartic-type lysosomal protease, ubiquitously expressed in all the cells and particularly expressed at a high level in the brain. In brain Cathepsin D assures neuronal homeostasis through degradation of unfolded or oxidized protein aggregates delivered to lysosomes via autophagy or endocytosis. In neurodegenerative disease, abnormal accumulation of neuronal proteins (e.g., the amyloid precursor, α-synuclein, and huntingtin) are due to diminished or abolished activity of Cathepsin D [66].

In order to confirm the alterations suggested by the DQBSA assay, we monitored cathepsin D maturation in the three cell lines through western blot analysis (Figure 2C). The ratio between the two immature forms and the 32-kDa mature form was strongly altered in *PARK2*-PD cells. Indeed, we observed a significant four-fold increase of immature forms in *PARK2*-PD cells compared to CTR1 and CTR2 controls, indicating that the pathway of Cathepsin D maturation is impaired in *PARK2*-PD cells (Figure 2D).

Altogether, these data strongly indicate that the lysosomal compartment is dysfunctional in *PARK2*-PD fibroblasts.

### 3.3. Autophagic Flux Is Impaired in PARK2-PD Fibroblasts

**Figure 3:**
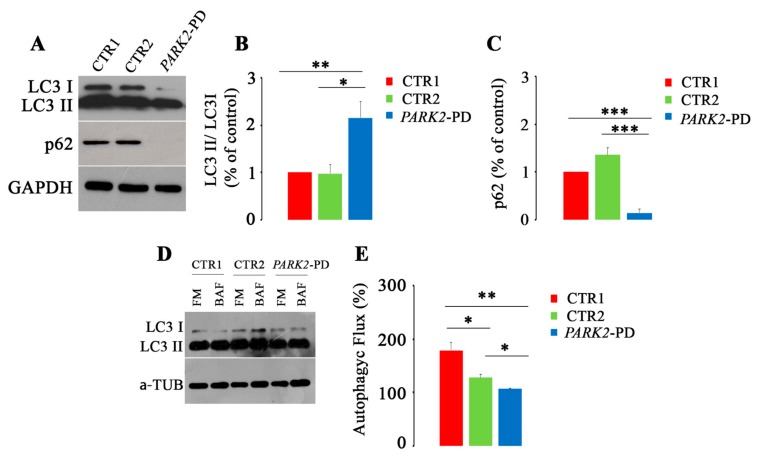
Alterations of autophagy in *PARK2*-PD cells. (**A**–**C**) Expression of LC3I, LC3II and p62 was evaluated through western blotting analysis. Densitometric analysis was performed, LC3I/ LC3II ratio was calculated and p62 expression was normalized against GAPDH. (**D**,**E**) Cells were incubated with (full medium) FM or (bafilomycin A) BAF and western blotting was performed for LC3 and α-tubulin. The autophagic flux was calculated as the ratio of LC3 between BAF and FM of the same sample. Data represent the mean ± SE of at least three independent experiments (* *p* ≤ 0.05, ** *p* ≤ 0.01 *** *p* ≤ 0.001).

In order to establish if the alterations detected in the endocytic pathway also affected the autophagic process, we decided to analyze through western blot analysis the expression of microtubule-associated protein light chain 3 (LC3) and p62, two known markers of autophagy [67]. Indeed, during the phase of elongation/nucleation of the phagophore, LC3 is cleaved to form LC3-I and subsequently conjugated to phosphatidylethanolamine (PE) to form LC3-II, localizing on isolation membrane and autophagosomes [48,68]. For this reason, the amount of LC3 reflects the number of autophagosomes and autophagy-related structures. Instead, p62 is directly bound to LC3 and selectively degraded during the autophagic process [69]. As shown in Figure 3A and in subsequently densitometric analyses (Figure 3B,C), *PARK2*-PD cells revealed a significant increase of the LC3II/LC3I ratio (about 2 fold) compared to controls and a significant reduction of p62 of about 85% and 120% compared to CTR1 and CTR2, respectively. 

The autophagic activity is not necessarily estimated through measurements of the LC3 amount because inhibition of autophagosome degradation also increases LC3 amount and LC3 can localize to non-autophagosomal structures, which are excluded by lysosomal turnover [70,71]. Furthermore, p62 degradation does not only indicate activation of autophagy because p62 is also involved in a number of other signaling pathways [72]. 

To this purpose, to better study autophagy in these cells, we have evaluated the autophagic flux and we found that both CTR2 and *PARK2*-PD showed an autophagic flux significantly impaired compared to CTR1 control cells (Figure 3D,E).

Altogether, these results indicate that in *PARK2*-PD fibroblasts autophagic flux is compromised.

### 3.4. Mitochondrial DNA Mutations and Biogenesis Dysfunction in PARK2-PD Fibroblasts

**Figure 4:**
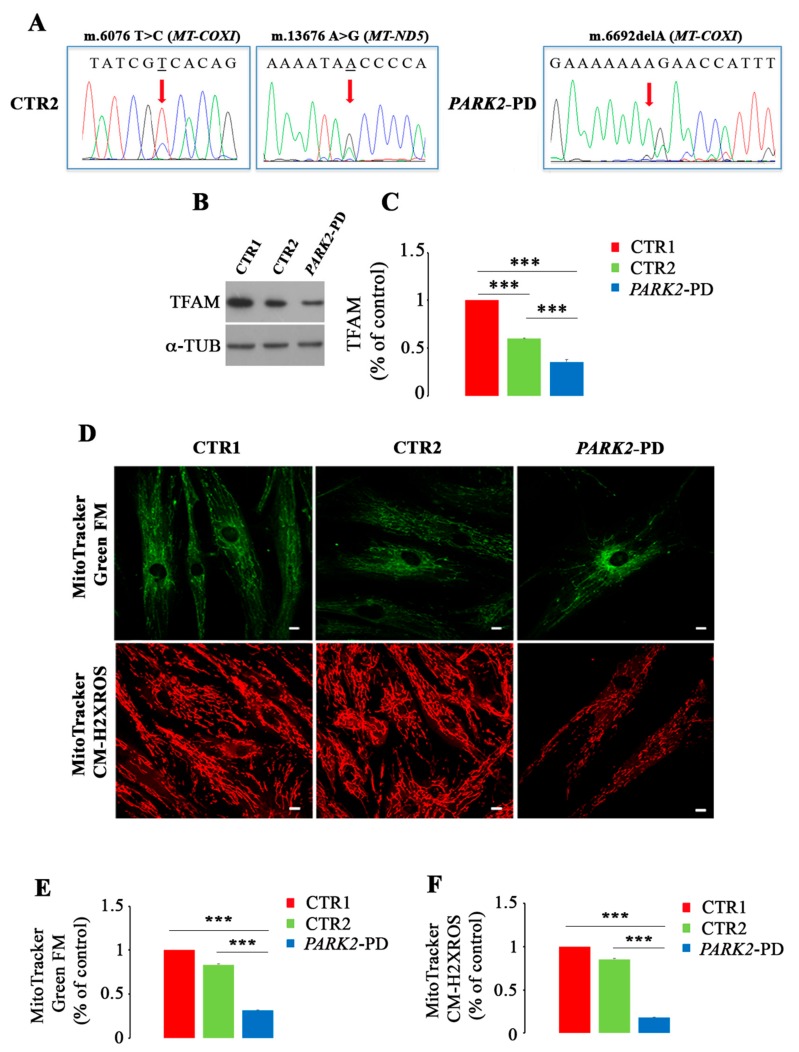
Mitochondrial defects in *PARK2*-PD cells. (**A**) Electropherograms of mitochondrial loci harboring mutations in CTR2 and *PARK2*-PD cells. Red arrows indicate the mutated and deleted bases. (**B**,**C**) Relative protein abundance of TFAM was assessed by western blotting and quantified by densitometry normalizing against α-tubulin. (**D**–**F**) Staining in live imaging of mitochondria with MitoTracker Green FM (green) and MitoTracker CM-H2XRos (red) in the three cell lines. Quantification of different intensities was performed with ImageJ software and represented in histograms (three independent experiments, ≥ 20 cells). Scale bar: 10 μm. Data represent the mean ± SE of at least three independent experiments (* *p* ≤ 0.05, ** *p* ≤ 0.01 *** *p* ≤ 0.001).

Mitochondrial impairment in *PARK2*-PD cells compared to CTR2 has been already described [39]. To further investigate this issue, we searched for genetic alterations in the mitochondrial DNA (mtDNA) as they could be one of the causes of mitochondrial dysfunction. Thus, we sequenced the whole mtDNA of CTR1, CTR2 and *PARK2*-PD cells. CTR2 and *PARK2*-PD cells displayed the same array of variants describing the two cells as belonging to the same haplogroup and providing a quality control to confirm the two subjects were of the same family. Interestingly, we found two heteroplasmic missense mutations in the mtDNA of CTR2 (m.6076T>C in *MT-COXI* and m.13676A>G in *MT-ND5*), predicted in silico as possibly damaging, and a heteroplasmic frameshift mutation in the mitochondrial genome of *PARK2*-PD (m.6692delA in *MT-COXI*) (Figure 4A). Since these mutations were detected at very early passages of both fibroblast lines, we exclude such mutations to be artifacts occurred subsequent to culturing, as it would take much longer for them to accumulate to the high loads of heteroplasmy that we here observed. Amino acid change, nucleotide variability and disease score for CTR2 mtDNA mutations are reported in Table 1.

Mitochondrial DNA mutations, mostly when they induce mitochondrial respiratory chain dysfunction, may trigger compensatory activation of mitochondrial biogenesis with the aim to increase mitochondrial turnover [73,74]. At the same time the presence in a cell of damaged/dysfunctional mitochondria may trigger autophagy with the aim to rid the cytoplasm of organelles with a deranged OXPHOS. Cells energy demand induces mitochondrial biogenesis and, among others, the Mitochondrial Transcription Factor A (TFAM), through its several functions, regulates such process [75]. Thus, we looked at TFAM expression in control and patient cells and we observed a significant downregulation of TFAM both in *PARK2*-PD and in CTR2 cells of about 65% and 40% compared to CTR1, respectively (Figure 4B,C). Also, TFAM was reduced in *PARK2*-PD of about 40% compared to CTR2 (Figure 4B,C), likely in agreement with the more damaging nature of the frameshift mutation in Complex IV, the bottleneck complex of the respiratory chain, and with the low expression of respiratory chain subunit previously reported [39].

To more deeply understand the mitochondrial phenotype in *PARK2*-PD cells, we performed a quantification of mitochondrial mass and of mitochondrial membrane potential using two different probes: MitoTracker Green FM, which stains mitochondria regardless of mitochondrial membrane potential and is an indicator of mitochondrial mass and MitoTracker Red CM-H2XROS, whose accumulation in mitochondria depends on mitochondrial membrane potential [42,43]. Using MitoTracker Green we observed a different intracellular distribution of mitochondria in *PARK2*-PD cell compared to CTR1 and CTR2 (Figure 4D). Indeed, in CTR1 and CTR2 mitochondria were uniformly distributed in the cytoplasm, while in PD cells these organelles were mainly clustered in the perinuclear region (Figure 4D). Moreover, intensity quantification of MitoTracker Green signal revealed a decreased staining of about 20% for CTR2 and of about 70% for *PARK2*-PD compared to CTR1, indicating a strong and significant reduction of the mitochondrial mass in patient cells (Figure 4E). In contrast, MitoTracker Red CM-H2XROS staining showed a similar disperse distribution of mitochondria in the cytoplasm of control and patient cells but a decreased staining for CTR2 and *PARK2*-PD. In fact, quantification using ImageJ software revealed a significant reduction of signal of about 20% and 70% in CTR2 and *PARK2*-PD, respectively (Figure 4F). These data indicate a reduction of the mitochondrial membrane potential in *PARK2*-PD cells.

These results demonstrate that the discovered mtDNA mutation is associated to mitochondrial dysfunction in *PARK2*-PD fibroblasts, and suggest that there is no compensatory activation of mitochondrial biogenesis as TFAM and other PGC-1α downstream target genes are downregulated or unchanged [39].

**Figure 5:**
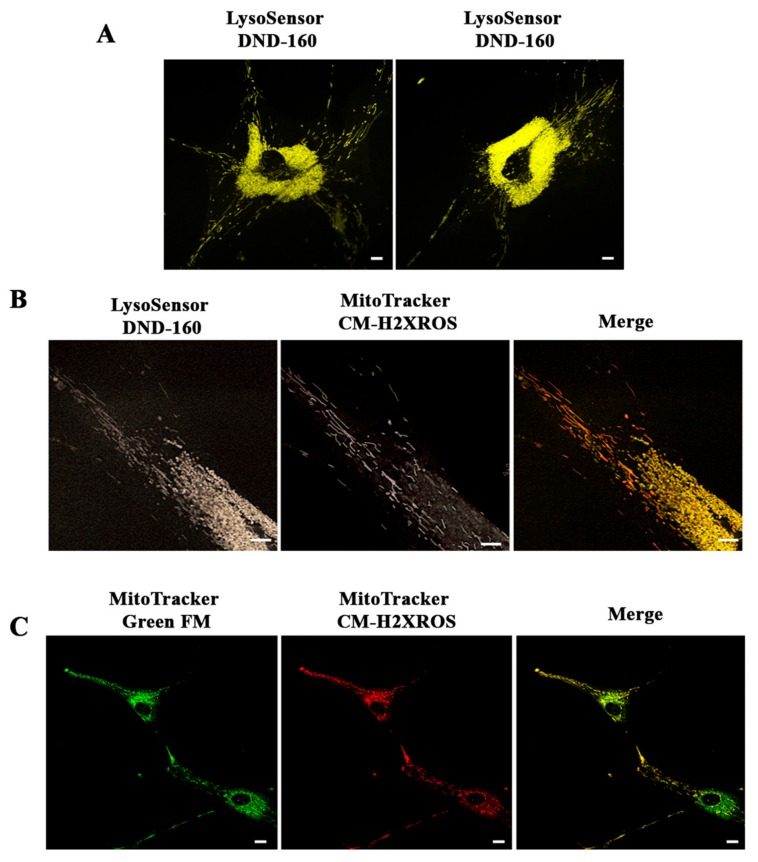
Altered intracellular distribution of mitochondria in *PARK2*-PD cells. (**A**,**B**) Mitochondrial and lysosomal staining in *PARK2*-PD cells was performed with LysoSensor DND-160 (yellow) and MitoTracker CM-H2XRos (red). In A two different *PARK2*-PD cells labeled with LysoSensor DND-160 are shown. In B a peripheral detail of a cell labeled with LysoSensor DND160 and MitoTracker CM-H2XROS is shown (**C**) Staining with MitoTracker Green FM (green) and MitoTracker CM-H2XRos (red) in *PARK2*-PD cells showing mitochondria in peripheral structures connecting cells suggesting mitochondrial transfer to neighboring cells. Scale bar: 10 μm.

Interestingly, after staining with LysoSensor DND-160 we observed, only in *PARK2*-PD cells, peripheral structures very similar to mitochondria. In Figure 5A, two examples of PD cells labeled with LysoSensor DND-160 are shown (Figure 5A). To clarify the nature of these structures, we performed double staining using LysoSensor DND-160 and MitoTracker Red CM-H2XROS and we were able to find a number of organelles labeled by both dyes, thus confirming that they are mitochondria (Figure 5B). 

Using MitoTracker Red CM-H2XROS and MitoTracker Green FM staining, in *PARK2*-PD cells, we could frequently observe mitochondria labeled with both dyes in peripheral structures similar to filopodia and connecting cells (Figure 5C). This suggests that *PARK2*-PD cells, in which both mitochondrial biogenesis and autophagic flux are blocked, may attempt to compensate for the energetic deficit through transfer of mitochondria. 

### 3.5. Premature Senescence in PARK2-PD Fibroblasts

We next investigated whether, along with dysfunction of lysosomal compartment, accumulation of damaged mitochondria would induce activation of cellular senescence. This mechanism is physiologically activated to prevent the propagation of damaged cells, but it is also associated with loss of function present in ageing and age-related diseases [76]. 

**Figure 6:**
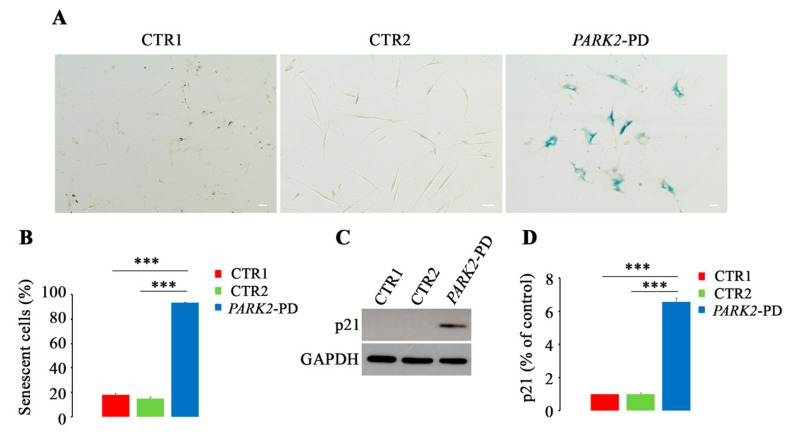
Premature senescence in *PARK2*-PD cells. (**A**,**B**). Senescence-associated β-galactosidase staining in CTR1, CTR2 and *PARK2*-PD cells. One representative experiment of three is shown. Magnification x4. Scale bar 75 μm. Senescent cells were counted and normalized on total amount of cells and represented in histograms. (**C**,**D**) Protein expression of p21 was evaluated by western blotting and abundance quantified by densitometric analysis normalizing against GAPDH. Data represent the mean ± SE, Standard Error of at least three independent experiments (* *p* ≤ 0.05, ** *p* ≤ 0.01 *** *p* ≤ 0.001).

A shift from oxidative phosphorylation to glycolysis, already described in *PARK2*-PD [39] and upregulation of lysosomal senescence-associated β-galactosidase [77,78] are characteristic changes of senescent cells which, unlike apoptosis, remain viable and metabolically active. Furthermore, it is known that dysfunctional mitochondria and increased number of lysosomes are important features of cellular senescence [78,79]. Thus, understanding whether the impairment of mitochondrial function, influencing lysosomal/autophagy function, triggers cellular senescence would allow linking the two cellular processes in *PARK2*-PD. Therefore, we decided to investigate the presence of senescent cells measuring activity of β-galactosidase at pH 6.0 by using in situ staining with the chromogenic substrate X-gal [54]. We performed the assay on CTR1, CTR2 and *PARK2*-PD cells at the same passage number (18) and we observed, as shown in Figure 6A, that PD fibroblasts were mostly senescent. Indeed, about 95% of *PARK2*-PD cells were β-gal positive while CTR1 and CTR2 populations contained only 18% and 15% of senescent cells, respectively (Figure 6B). 

To validate this result, we also analyzed the expression of CDKi (cyclin-dependent kinase inhibitor) p21 as senescent cells arrest permanently their cell cycle, through regulation by p16INK4A and p53-p21-RB (retinoblastoma). The cell cycle arrest is due initially to upregulation of p21, induced by increased expression of p53 and then becomes permanent through inhibition of CDK4 and CDK6 by p16INK4A and consequent RB hypophosphorylation, blocking entry to the S phase [80]. As shown in Figure 6C, we observed upregulation of p21 only in *PARK2*-PD cells with a statistically significant increase about of 6 fold compared to CTR1 and CTR2 cells.

These results suggest that the block of mitochondrial turnover, inhibiting generation of new functional mitochondria, and the alterations of autophagic flux, inhibiting the efficient elimination of damaged organelles, may determine premature senescence in *PARK2*-PD fibroblasts.

### 3.6. Inhibition of Complex I Activity Induce Lysosomal Dysfunction

**Figure 7:**
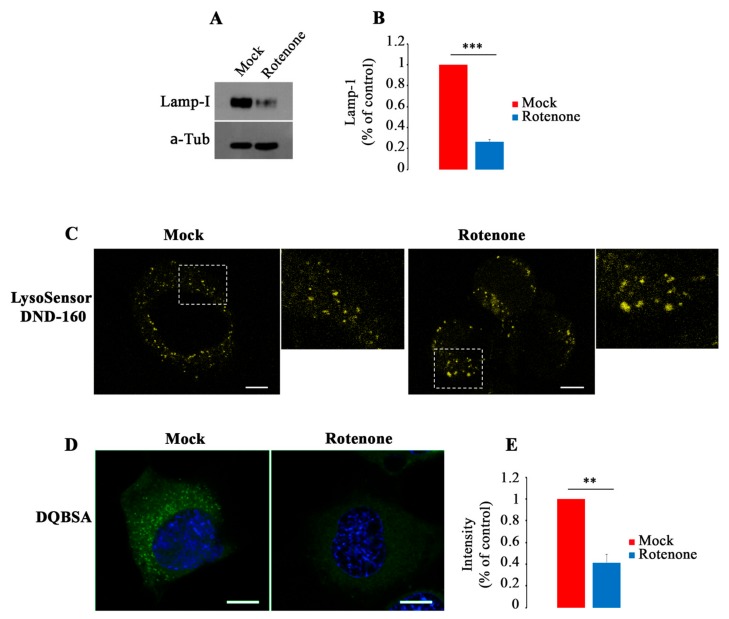
Rotenone induces lysosomal dysfunction in NCS34 cells. (**A**,**B**) Abundance of Lamp-I was evaluated by Western blot analysis and quantified by densitometric analysis normalizing against α-tubulin. (**C**) Staining with LysoSensor DND-160 (yellow) in Mock and Rotenone-treated (100 nM) cells showing increased staining of enlarged lysosomes after treatment. Dashed boxes represent zoomed areas on the right. Scale bar: 10 μm. (**D**,**E**) Cells were incubated in the presence of Green DQ-BSA and nuclei were labeled with DAPI (blue). Fluorescence intensity was quantified by ImageJ software. Scale bar: 10 μm. Data represent the mean ± SE of at least three independent experiments (* *p* ≤ 0.05, ** *p* ≤ 0.01 *** *p* ≤ 0.001).

In order to understand if treatment with Rotenone, an inhibitor of complex I activity, might induce similar lysosomal alterations as observed in *PARK2*-PD fibroblasts, we treated NSC34 cells with this drug and we analyzed structural and functional changes in lysosomes. Interestingly, in treated cells, we found a significant decrease of Lamp-1 expression (Figure 7A,B), a significant increase of stained lysosomes with LysoSensor DND-160 (Figure 7C) and a significant reduction of Green DQBSA (Figure 7D,E).

These results indicate that the inhibition of CI activity, inducing ROS production, may be the cause of alterations in biogenesis, morphology and function of lysosomes.

## 4. Discussion

In this paper, we show that impairment of autophagy and lysosomal function in *PARK2*-PD fibroblasts is associated with mitochondrial defects, determining accumulation of non-functional mitochondria and cellular senescence as a result of mitochondria-lysosomal cross-talk.

Our hypothesis arises from the observation that patient cells grew very slowly and were characterized by mitochondrial dysfunction [39]. Indeed, it was previously demonstrated that, in these cells, decrease of CI and CIV activity and defective OXPHOS capacity causes impairment of mitochondrial function and compensatory increase of glycolysis [39]. Moreover, this is accompanied by a remarkable dysfunction of PGC-1α, the master regulator of mitochondrial biogenesis, unable to activate downstream target genes [39]. For these reasons, we searched for mtDNA mutations in parental control and *PARK2*-PD cells. We found a frameshift mutation in *MT-COXI* in *PARK2*-PD fibroblasts, possibly contributing to the observed mitochondrial impairment, and mutations in *MT-COXI* and *MT-ND5* in the healthy parental control (CTR2). Although unexpected, these slight differences in genotype might be as a result of the continuous shifts in heteroplasmy that unfixed mtDNA mutations undergo, particularly in the context of cells with an impaired mitochondrial turnover such as these. Deep sequencing analyses are warranted to understand whether the same mutations indeed occur in both subjects analyzed. Indeed, they could be at too low heteroplasmic levels to be detected by Sanger sequencing or they could represent somatic events occurred in the connective tissue of single individuals.

Mitochondrial respiratory complexes are organized in supramolecular associations to form “super complexes” with catalytic enhancement, substrate channeling, and stabilization of CI by CIII in mammalian cells [81]. Structural dependence between these complexes was confirmed by dramatic loss of CI as result of the absence of assembled CIII [82,83]. Moreover, CIV is required for assembly and stability of CI [84]. Finally, interaction between CI, CIII and CIV in pre-respirasome structures ensures complete assembly of CI [85]. According to these data, the mtDNA mutation in *MT-COXI* described in *PARK2*-PD cells, in agreement with the low protein expression described in [39], might have an impact on the development of the Parkinsonism, as in this case CIV may be decreased in amount and availability to interact with CI for the proper operation of substrate channeling. Indeed, we confirmed mitochondrial impairment activity through labeling with the MitoTracker Red CM-H2XROS fluorescent dye, whose accumulation in mitochondria depends on mitochondrial membrane potential, and we observed reduced mitochondrial mass and perinuclear clustering, when we stained with MitoTracker Green FM dye, which stains mitochondria regardless of mitochondrial membrane potential. Interestingly, it was reported that perinuclear clustering of mitochondria is associated with accumulation of nuclear ROS in hypoxic condition [86]. Thus, the high level of ROS, OXPHOS dysfunction and activation of compensatory glycolytic pathway measured in *PARK2*-PD cells, could mimic the cellular response to hypoxic conditions [39]. The missense mutations found in CTR2, on the other hand, although likely below the threshold for a clear-cut pathogenic effect, may account for the intermediate phenotype that we observe (Figure 4B–F). In any case, whether mitochondrial mutations in *PARK2*-PD cells are a direct cause of PD pathogenesis or a secondary consequence of an ongoing mitochondrial dysfunction is still not clear, as previously observed [87].

Mitochondrial turnover is guaranteed by balance between mitochondrial biogenesis and autophagy elimination of damaged organelles. Indeed, mtDNA mutations induce compensatory activation of mitochondrial biogenesis to increase the number of mitochondria in order to compensate mitochondrial deficit [73]. The health of a mitochondrial population is assured also by efficient clearance mechanisms to remove dysfunctional mitochondria from the organelle pool, a mechanism particularly important in post-mitotic cells, such as neurons, characterized by high energetic demand and low regenerative capacity where mitochondrial dysfunction may cause cell death [88]. In this scenario, autophagy or selective degradation of damaged mitochondria, known as mitophagy, are key cellular mechanisms to remove dysfunctional mitochondria. The most studied pathway to remove damaged mitochondria is the PINK1-Parkin dependent-mitophagy [89]. Previously, it was demonstrated that *PARK2*-PD fibroblasts, carrying a compound heterozygous deletional mutation of *PARK2* gene (del exon2/3 and del exon 3), have lost full-length parkin protein compared to CTR2, which displayed only heterozygous deletion in exon 3. However, a growing body of evidence has shown that the mitophagy pathway is not unique and that there are additional regulatory processes for the clearance of damaged mitochondria, most of which require functional lysosomes [90]. Thus, our data indicating that in patient cells lysosomes are not fully functional support the accumulation of impaired mitochondria. 

Recently, the importance of mitochondria-lysosomes contacts has been highlighted in the maintenance of cellular homeostasis [91]. Interestingly, formation and stabilization of contacts between the two organelles was promoted by GTP-bound Rab7 and inhibition of Rab7 hydrolysis leaded to abnormally large lysosomes and extended duration of mitochondria-lysosomes contacts [91]. Here, we observed an increased number of enlarged lysosomes in *PARK2*-PD fibroblasts using LysoSensor and LysoTracker dyes. We think that the increased lysosomal fluorescent staining is due to accumulation of lysosomes, suggesting an impairment of the late endocytic pathway. Indeed, Cathepsin D maturation, DQBSA assay and autophagic flux evaluation showed dysfunctional lysosomal compartment in *PARK2*-PD fibroblasts. 

Mitochondrial dysfunction was described as responsible of lysosomal alterations [58]. Indeed, impairment of ATP production affects also V-ATPase (responsible for acidification of the endocytic pathway), as ATP produced by compensatory activation of glycolysis could be not sufficient to guarantee also this function. Surprisingly, not only *PARK2*-PD cells but also CTR2 cells display downregulation of ATP6V1G1 expression, alterations of Cathepsin D maturation and inhibition of the autophagic flux, but in CTR2 cells mitochondrial biogenesis is active and might ensure a healthy mitochondria population compensating the energetic deficit. 

Interestingly, we observed peripheral mitochondria-like structures labeled with LysoSensor DND-160 in *PARK2*-PD cells. Using MitoTracker Red CM-H2XROS we confirmed that mitochondria were also present at cell periphery, suggesting that the respiratory chain deficit may induce partial dissipation of mitochondrial proton gradient determining their acidification and making them recognizable by the lysosomal dye. Cell-to-cell transfer of mitochondria, known as horizontal mitochondrial transfer, is a phenomenon described in human cells [92]. To overcome mitochondrial failure and ensure cell survival, cells with mitochondrial dysfunction are able to acquire new mitochondria from donor cells. In fact, it was demonstrated that mitochondria and/or mtDNA can move between cells and rescue aerobic respiration in dysfunctional acceptor cells [92]. Mitochondrial transfer is achieved though several mechanisms, including tunneling nanotubes, microvesicles, channels and exoxomes [93,94,95,96]. Block of mitochondrial turnover, due to dysfunctional mitochondrial biogenesis and autophagic flux, could induce *PARK2*-PD cells to try to compensate the energetic deficit through transfer of mitochondria or in the attempt to get rid of dysfunctional ones. Actually, the reason for cell-to cell mitochondrial transfer is still unclear, but it was also hypothesized that it could be a mechanism employed when degradation pathways are compromised, in order to maintain the cell fit [97]. 

*PARK2*-PD fibroblasts are characterized by a very slow growth and a peculiar cellular morphology, as previously published [55]. Moreover, these cells need a high cellular density to proliferate. As pathological features of *PARK2*-PD are present also in primary fibroblasts from patients [98], understanding the growth arrest mechanisms in *PARK2*-PD cells could help to explain dopaminergic neurons loss. In this work, we demonstrated that PD fibroblasts arrested their growth through a mechanism leading to premature senescence. Large and numerous lysosomes and increased levels of β-galactosidase have been described in senescent cells [99], Also, mitochondrial dysfunction, changes in mitochondrial morphology and ROS production are characteristics of the senescent phenotype [99]. All these features are present in *PARK2*-PD cells.

It is important to emphasize that skin fibroblasts represent a good model to study PD pathogenesis harboring defined mutations and the cumulative cellular damage of the patients. In fact, it has been proposed that skin fibroblasts from PD patients are a good cellular system to model the disease, being able to reproduce several of the molecular alterations occurring in this neuropathology [100,101,102,103,104]. Indeed, according to polygenic predisposition and environmental etiopathology, skin fibroblasts comprise the chronological and biological aging of the patients [100]. Furthermore, procedures to perform skin biopsies are minimally invasive and allow the possibility to obtain primary cultures of self-propagating cells with patient’s genomic background without additional genetic manipulation. Elucidation of altered pathway in skin fibroblasts from PD patients can contribute to understand in vivo mechanism of neurodegeneration and plays a significant role in future PD diagnosis and in cell-replacement therapy.

Finally, it is known that rotenone is among the possible causes of sporadic (idiopathic) PD, inducing mitochondrial impairment, ROS production, protein oxidation increase and dopaminergic neurodegeneration [105,106]. Moreover, acute exposure to rotenone determines, as consequence of mitochondrial stress, activation of AMPK signaling, inhibition of mTOR and TFEB with block of lysosomal biogenesis [31]. Furthermore, it was demonstrated that lysosomal impairment can be due to mitochondrial ROS production [34]. According to this, in NCS34 cells treated with Rotenone we found a decrease of Lamp-I expression, indicating inhibition of lysosomal biogenesis, and dysfunctional lysosomes, as demonstrated by inhibition of Cathepsin D maturation and of DQ-BSA degradation. Therefore, we hypothesize that mitochondrial perinuclear clustering, observed in *PARK2*-PD cells, could induce oxidative insult to autophagosomes, which are concentrated in perinuclear region, determining also autophagic and lysosomal impairment. 

In conclusion, we have characterized *PARK2*-PD fibroblasts discovering impairment of both mitochondrial and lysosomal functions possibly causing growth arrest and senescence. We believe to have elucidated new aspects of pathogenesis and neurodegenerative progression in juvenile form of PD with *PARK2* mutation showing that premature senescence could be accounted for or contribute to dopaminergic neuron loss as a result of cross-talk between dysfunctional mitochondria and lysosomes. Although further investigations are needed to better understand PD pathogenesis, both of familiar and idiopathic forms, our observations could aid to better explain the role of mitochondria, oxidative stress, lysosomal and autophagy dysfunction in the progression of this disease with the ultimate goal of identifying pharmacological therapies to prevent and/or arrest dopaminergic neurodegeneration. 

## Figures and Tables

**Table 1:** Analysis of CTR2 mtDNA mutations.

Variant	Gene	Amino Acid Change	Polyphen2 Score	Nucleotide Variability
m.6076T>C	*MT-COXI*	V58A	0.783	0.000277
m.13676A>G	*MT-ND5*	D447S	0.51	0.0005
